# Drug- and Toxin-Induced Opsoclonus – a Systematized Review, including a Case Report on Amantadine-Induced Opsoclonus in Multiple System Atrophy

**DOI:** 10.5334/tohm.832

**Published:** 2024-05-08

**Authors:** Hugo Cannilla, Maria Messe, François Girardin, François-Xavier Borruat, Julien F. Bally

**Affiliations:** 1 University of Lausanne, Lausanne, Switzerland; 2 Geneva University Hospitals and University of Geneva, Geneva, Switzerland; 3 Division of Clinical Pharmacology, Department of Laboratory Medicine and Pathology, Lausanne University Hospital & University of Lausanne, Lausanne, Switzerland; 4 Department of ophthalmology, Jules Gonin Eye Hospital, University of Lausanne, Lausanne, Switzerland; 5 Service of Neurology, Department of Clinical Neurosciences, Lausanne University Hospital & University of Lausanne, Lausanne, Switzerland

**Keywords:** Opsoclonus, Opsoclonus-Myoclonus Syndrome, Drug-induced, Multiple system atrophy, Drug Adverse effects

## Abstract

**Background:**

Opsoclonus is a rare disorder characterized by conjugate multidirectional, horizontal, vertical, and torsional saccadic oscillations, without intersaccadic interval, resulting from dysfunction within complex neuronal pathways in the brainstem and cerebellum. While most cases of opsoclonus are associated with autoimmune or paraneoplastic disorders, infectious agents, trauma, or remain idiopathic, opsoclonus can also be caused by medications affecting neurotransmission. This review was prompted by a case of opsoclonus occurring in a patient with Multiple System Atrophy, where amantadine, an NMDA-receptor antagonist, appeared to induce opsoclonus.

**Methods:**

Case report of a single patient and systematized review of toxic/drug-induced opsoclonus, selecting articles based on predefined criteria and assessing the quality of included studies.

**Results:**

The review included 30 articles encompassing 158 cases of toxic/drug-induced opsoclonus. 74% of cases were attributed to bark scorpion poisoning, followed by 9% of cases associated with chlordecone intoxication. The remaining cases were due to various toxics/drugs, highlighting the involvement of various neurotransmitters, including acetylcholine, glutamate, GABA, dopamine, glycine, and sodium channels, in the development of opsoclonus.

**Conclusion:**

Toxic/drug-induced opsoclonus is very rare. The diversity of toxics/drugs impacting different neurotransmitter systems makes it challenging to define a unifying mechanism, given the intricate neuronal pathways underlying eye movement physiology and opsoclonus pathophysiology.

## Introduction

Visual fixation is crucial for stabilizing the eyes on the target of interest and is essential for optimal visual performance, especially using the retinal fovea in humans. A steady fixation is achieved through various mechanisms, including the pursuit system (if the visual target is moving), the vestibulo-ocular pathway (if the subject’s head is in motion), the stability of the eye in space (controlled by the brainstem neural integrator), and the inhibition of the saccadic system [1].

Spontaneous undesired interruptions of fixation can result from two main causes: (i) a slow drift from fixation known as nystagmus or (ii) abnormal saccades that rapidly move the eyes away from the target, leading to a spectrum of spontaneous abnormal eye movements [1]. This spectrum includes square wave jerks, ocular flutter and opsoclonus. Square wave jerks are characterized by a horizontal saccade followed by a pause and then another horizontal saccade that brings the eye back to its primary position [2]. Both ocular flutter and opsoclonus are high frequency (15–25 Hz) involuntary saccade with no intersaccadic interval, primarily differing in their direction. While ocular flutter involves solely a horizontal component, opsoclonus encompasses movements in multiple planes, including horizontal, vertical, and torsional directions [1,2,3]. These movements are frequently sizable enough to be visible during a clinical examination, without the aid of any instruments. However, opsoclonus and ocular flutter can manifest with extremely small amplitudes, rendering them imperceptible to the naked eye. Their detection may require the use of fundoscopy, examination at the slit lamp, or video-oculographic recordings [1,2].

Another definition by Margolin et al. emphasizes the chaotic nature of these eye movements: “Opsoclonus is an oculomotor dyskinesia characterized by rapid, repetitive conjugate eye movements that are involuntary, arrhythmic, chaotic, and multidirectional (horizontal, vertical, and torsional components) without intersaccadic intervals” [3]. The term “opsoclonus” was first used by Orzechowski, a Polish neurologist, in 1913 to describe a patient with “disorganized conjugate eye movement” [4]. Opsoclonus is frequently associated with myoclonus in the Opsoclonus-Myoclonus Syndrome (OMS) and/or ataxia in the Opsoclonus-Myoclonus-Ataxia Syndrome (OMAS), leading to complaints of visual blur and oscillopsia due to the high-frequency and large-amplitude ocular oscillations.

**Figure 1:**
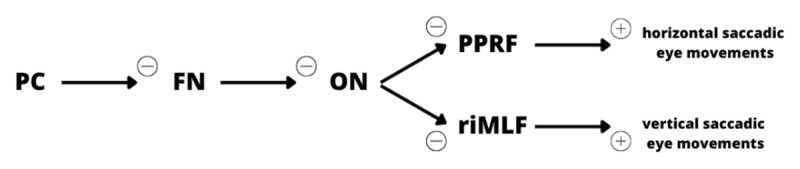
Neuronal structures and pathways implicated in physiologic saccadic eye movements, putatively implicated in the pathophysiology of opsoclonus. PC: purkinje cells; FN: fastigial nucleus; ON: omnipause neurons; PPRF: parapontine reticular formation; riMLF: rostral interstitial nucleus of the medial longitudinal fasciculus.

Although there is currently no scientific consensus on the underlying pathophysiology of opsoclonus, three main theories have been proposed (Figure 1).

1. The pontine theory suggests that damage to the omnipause neurons (ON) in the pontine nucleus raphe interpositus leads to saccadic intrusions. Normally, these ON inhibit the burst cells in the paramedian pontine reticular formation (PPRF) and rostral interstitial nucleus of the medial longitudinal fasciculus (riMLF) which are responsible for initiating saccades. Therefore, damage to the ON results in uninhibited burst cell activity and involuntary ocular saccades. However, no neuropathological evidence has definitively supported this theory, and lesions in the PPRF have been associated with slow saccades rather than abnormal oscillatory saccades. Autopsy findings have also failed to demonstrate consistent histopathologic changes in the omnipause cells of most opsoclonus patients [1,3,5,6,7,8].
2. The brainstem theory proposes that opsoclonus arises from alterations in the synaptic membrane properties of ON onto burst cells, making the latter susceptible to excessive post-inhibitory rebound excitation or unresponsive to efficient ON inhibition. However, clinical evidence supporting this theory is lacking, and synaptic membrane changes would typically result in smaller amplitude saccadic oscillations compared to those observed in opsoclonus [1,3,5,6,8].
3. The cerebellar theory suggests that dysfunctional cerebellar Purkinje cells fail to inhibit the fastigial nucleus (FN) in the cerebellum, which action is to inhibit the ON; consequently, ON are strongly inhibited and unopposed burst cells fire, leading to subsequent opsoclonus. Functional magnetic resonance imaging (fMRI) studies have confirmed increased activation of the FN, and positron emission tomography/computed tomography (PET/CT) scans have demonstrated abnormal Purkinje cell function. Additionally, a case of OMS with myoclonic epilepsy revealed the presence of a heterozygous missense mutation and a large deletion in the potassium channel tetramerization domain containing 7 (KCTD7) has been reported. The channel in question shows widespread expression in the cerebellum, which strengthens the idea of the cerebellum’s involvement in the underlying mechanisms of oscillatory saccades. This theory is further supported by histopathological evidence demonstrating damage to afferent projections to the fastigial nucleus [1,3,5,6,8,9,10,11].

Opsoclonus exhibits a broad range of etiologies including paraneoplastic, autoimmune, infectious, idiopathic, traumatic brain injury and drug-induced causes [1,2,5]. Auto-immune and paraneoplastic causes are frequent, with both humoral and cell-mediated immune mechanisms. In adults, paraneoplastic opsoclonus has been associated with various neoplasia, accompanied by numerous antineuronal antibodies, the list of which is beyond the scope of this review. However, despite advancements in identifying these autoantibodies, most of the patients are seronegative for known antineuronal antibodies, and a definitive diagnostic immunological marker is yet to be established [2,3,5,8,9].

In children, paraneoplastic syndromes arising from neuroblastic tumors such as neuroblastoma, ganglioneuroblastoma, and ganglioneuroma are the most common causes of opsoclonus and OMS [2,3]. Interestingly, patients with neuroblastoma who experience additional opsoclonus often have a more favourable prognosis [5]. This improved prognosis may be attributed to the early manifestation of opsoclonus, which allows for earlier detection and prompt initiation of treatment.

Auto-immune diseases such as multiple sclerosis, coeliac disease or sarcoidosis can occasionally present with opsoclonus. Other potential causes of opsoclonus include various central nervous system infections. Enterovirus, West Nile, Lyme, Mumps, HIV, Malaria, Dengue, and Zika are among the reported infectious causes; however, frequently, a specific infection cannot be identified [2,5]. It is essential to note that a substantial number of patients who experience isolated opsoclonus have an idiopathic origin for the condition.

In rare instances, opsoclonus or OMS can be induced by specific drugs or toxins. Despite some understanding of the putative pathophysiology, there is a lack of comprehensive reviews that thoroughly explain the toxic/drug-induced causes of these oculomotor abnormalities and their underlying mechanisms.

Recently, we encountered a patient who developed drug-induced opsoclonus resulting from amantadine administration, which appears to be a hitherto unreported side effect of this medication. This case prompted us to conduct an extensive literature search to gain a better understanding of the range of toxins or drugs that have the potential to induce opsoclonus. Furthermore, we aimed to identify the possible mechanisms that lead to toxin/drug-induced opsoclonus.

## Methods

1. Case report of a patient with opsoclonus induced by amantadine (challenge-rechallenge)
2. Systematized review of the literature searching for all reported cases of either toxic- or drug-induced opsoclonus, Opsoclonus-Myoclonus Syndrome (OMS) or Opsoclonus-Myoclonus-Ataxia Syndrome (OMAS). We included all types of studies, such as case reports and original articles, to ensure comprehensive coverage.

We conducted a systematized search on the PubMed and Embase databases using two customized search algorithms. Our strategy included a combination of general terms and Medical Subject Headings (MeSH) terms related to opsoclonus, its etiology, drug effects, adverse effects, chemically induced reactions, side effects, and toxicity. The search was conducted up to October 18, 2022, to gather relevant articles for our review.

After removing duplicate articles, we initially obtained a total of 826 articles from both databases. We conducted a screening process by reviewing the titles and, if necessary, the abstracts of these articles. We included articles in our review if they mentioned a toxic/drug-induced cause of opsoclonus, OMS, or OMAS. We excluded articles that focused on paraneoplastic, immunologic, or infectious/parainfectious causes, as well as review articles that did not provide new cases and non-English articles.

**Figure 2:**
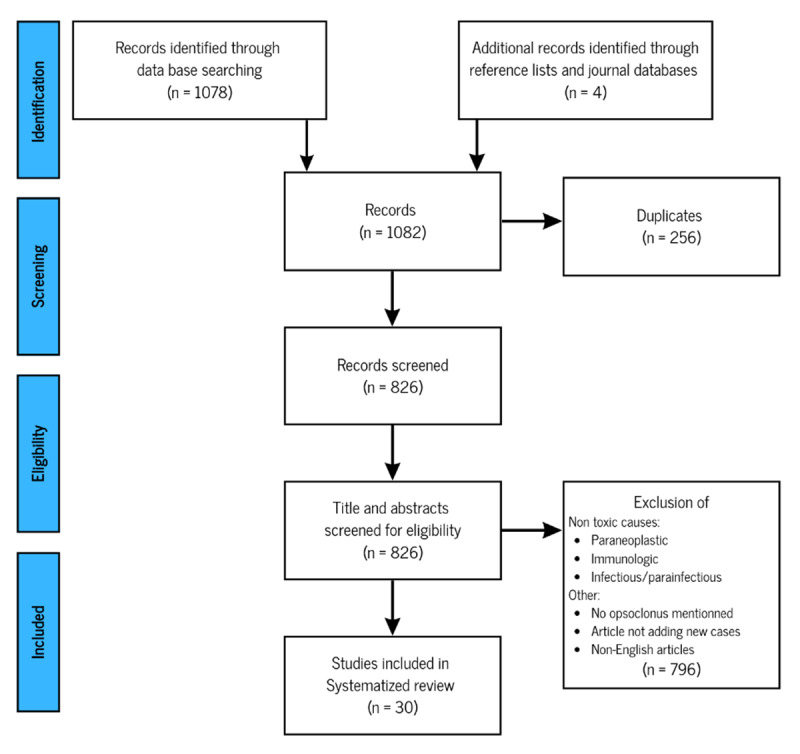
Algorithm of articles inclusion/exclusion.

During the screening process, we also checked the bibliography of each included article to identify any additional relevant studies, resulting in the inclusion of four more articles. Overall, our systematized review included a total of 30 articles that were deemed relevant for our investigation (Figure 2).

However, certain drugs reported to cause opsoclonus by Wong et al., 2007 [5] were not included due to either a lack of detailed description or a description that did not fit with the definition of opsoclonus [12].

The full text of each article was thoroughly reviewed, and detailed information from each study was recorded in an Excel file. Additionally, a separate file was created to classify the pharmacological details of each case. In this file, we assessed the imputability of the drug using the Naranjo score [13], which is a standardized tool commonly used by pharmacologists to determine the likelihood of a drug being the cause of an adverse event. The data collection processes provided us with a comprehensive overview of adverse events related to drug-induced or toxin-induced opsoclonus or OMS.

In our analysis, we focused on discussing the most relevant drugs associated with opsoclonus. These drugs were identified based on two criteria: (i) if the number of reported cases exceeds one (≥2), indicating a recurrent association, and (ii) drugs with a high likelihood of causing opsoclonus, as indicated by a Naranjo ≤6.

We made a deliberate decision not to include literature without peer-review, such as those referring to unpublished or non-peer-reviewed sources. While this approach ensures the reliability of the included studies, it is important to acknowledge that the omission of grey literature may have led to potentially relevant information not being captured.

A new search was performed on January 22^nd^, 2024, searching for “drug- and toxin-induced ocular flutter”, as opsoclonus and ocular flutter are virtually the same, the latter differing from the former by the solely horizontal direction of the saccadic bursts [1].

## Results

### Case report

A 67-year-old male patient visited our ambulatory clinic in November 2019 for a routine follow-up related to his parkinsonian and cerebellar syndrome, which was suggestive of probable multisystemic atrophy with parkinsonian predominance (MSA-P) since 2015. Upon retrospective analysis of his clinical picture, he fulfilled the 2022 Movement Disorders Society criteria for clinically established MSA [14], notably including a normal MIBG cardiac scintigraphy.

Due to the gradual progression of gait and balance difficulties, the patient’s treatment was adjusted, and amantadine was added (100mg/day for two weeks, then 100mg twice a day) to his existing prescription of levodopa/benserazide (Madopar^®^) 200/50mg four times daily. A few weeks later, the patient and his wife reported the occurrence of uncontrolled eye movements happening every evening, resulting in disturbed vision. During the follow-up visit, we personally observed and recorded sudden, multidirectional, paroxysmal, conjugate movements of both eyes in multiple directions, lasting for approximately 30 seconds, consistent with opsoclonus (Video 1).

**Video 1:** Opsoclonus happening during an outpatient visit when the patient was on 200 mg of amantadine.

The patient and his wife reported that the involuntary eye movements began on the third week after starting amantadine, coinciding with the dosage increase from 100 mg to 200 mg daily. In response to this new symptom, we promptly discontinued the use of amantadine and conducted a comprehensive medical evaluation to determine the underlying cause of the opsoclonus.

Laboratory tests were performed, revealing normal renal and hepatic function, with no electrolyte imbalances detected. Immunological investigations (FAN, ANCA) and infectious screenings (HIV, borreliosis, syphilis, hepatitis, tuberculosis, Epstein-Barr virus, and West-Nile virus) yielded negative results. Additionally, specific antibodies associated with paraneoplastic autoimmune encephalitis were not detected in the cerebrospinal fluid (AMPA-R, Amphiphysin, AGNA-1, ANNA-1-2-3, CASPR2, CRMP-5/CV2, DPPX, GABA-B, GAD65, GFAP, LgI-1, mGluR1, NMDA, PCA). A specialized analysis for Whipple’s disease also showed no evidence of the condition (Tropheryma whipplei DNA was negative in EDTA-blood research).

Lumbar puncture did not reveal any signs of infection, whether viral or bacterial. Brain MRI showed global cerebral atrophy, particularly pronounced in the posterior fossa and cerebellar peduncles, which is consistent with a diagnosis of MSA. However, there were no signs of tumor or white matter lesions. Whole-body PET-CT imaging did not reveal any tumoral cause, and the electroencephalogram showed normal activity. Despite the comprehensive etiological evaluation, a specific cause for the opsoclonus could not be identified. Following the discontinuation of amantadine, the episodes of opsoclonus rapidly disappeared.

During a follow-up visit six months later, the patient did not report any recurrence of opsoclonus since the discontinuation of amantadine, nor did he experience any new neurological symptoms. However, he still complained of gait problems. With the patient’s consent, we decided to reintroduce amantadine at a lower dose (100 mg daily). Three days later, the patient experienced oscillopsia, and the opsoclonus reappeared. Amantadine was immediately stopped again, and the opsoclonus disappeared once more. Following this challenge-rechallenge test, it became evident that amantadine was the cause of the opsoclonus. As a result, amantadine was definitively excluded from the patient’s medication regimen, and the opsoclonus did not recur during the three years that followed.

Based on the patient’s medical history, physical findings, negative ancillary examinations, and the positive challenge-rechallenge test with amantadine, we concluded that the opsoclonus resulted from a side-effect of the medication.

### Literature review

We encountered 30 articles comprising 158 cases, which reported various drugs and toxins leading to opsoclonus. Among these, the vast majority of cases (117 instances, 74%) were attributed to bark scorpion poisoning, followed by 15 cases (9%) associated with chlordecone intoxication. The remaining 26 cases involved exposure to 19 different drugs or toxic substances (Table 1). In the following discussion, we focus on drugs with a substantial imputability, i.e., those with a Naranjo score of ≥6, or reported in more than one subject, which are highlighted in bold in Table 1. Other drugs that potentially could trigger opsoclonus but have a lower certainty of causality or limited supporting literature are also listed in Table 1 without further discussion.

**Table 1:** List of toxic/drugs causing opsoclonus classified by number of cases (n = ) and in the order of appearance in the article.


DRUG/TOXIC	PUTATIVE IMPLICATED SYSTEM	REFERENCE	NUMBER OF CASES (PER MECHANISM OF ACTION)	n	NARANJO SCORE

**bark scorpion poison** (c. sculpturatus)	sodium channel inactivation, increased release of norepinephrine and acetylcholine	[15]	n = 117	n = 84	6
	
		[16]		n = 33	7

**chlordecone** (or kepone, merex, curlone)	mitochondrial Na+/K+ ATPase inhibition	[17]	n = 15	n = 15	5
	
		[18]			6
	
		[19,20]			6

**diphenhydramine (DPH)**	anticholinergic & antihistaminic H1	[23]	n = 3	n = 1	4
	
		[24]		n = 1	7
	
		[25]		n = 1	5

**chlorpyrifos** (organophosphate)	cholinergic	[26]	n = 3	n = 1	6
		
**malathion** (organophosphate)		[27]		n = 1	7
		
**parathion and methyl-parathion** (organophosphates)		[28]		n = 1	6

**lamotrigine** (associated with valproate – probably not implicated)	various mechanisms: voltage-gated sodium channel blocker or GABAergic	[32]	n = 3	n = 1	6
		
**phenytoin associated and diazepam, versus barbiturate withdrawal**		[33]		n = 1	6
		
**phenytoin** (associated with levetiracetam – probably not implicated)		[34]		n = 1	5

**cocaine**	serotoninergic & dopaminergic & noradrenergic (reuptake blockage)	[36]	n = 2	n = 1	7
	
		[37]		n = 1	7

**cyclosporine A**	dopaminergic	[7]	n = 2	n = 1	5
	
		[38]		n = 1	6

**toluene** (methylbenzene)	GABAergic, glutamatergic, and cholinergic	[41]	n = 2	n = 1	5
	
		[42]		n = 1	6

**lithium associated with haloperidol**	glycinergic & dopaminergic	[45]	n = 2	n = 2	4

**amantadine** (our case report)	glutamatergic		n = 1	n = 1	7

**phencyclidine (PCP)**	glutamatergic & GABAergic	[9]	n = 1	n = 1	6

**solanum torvum berries** (or turkey berries, susumber, aubergine sauvage)	cholinergic	[49]	n = 1	n = 1	6

**thallium**	potassium interference (Na+/K+-ATPase)	[52]	n = 1	n = 1	6

**cefepime**	GABAergic	[54]	n = 1	n = 1	6

adenovector viral (AVV) ChAdOx1 vaccine (AZD1222) (AstraZeneca)	unclear	[63]	n = 1	n = 1	3

carbon monoxide	unclear	[64]	n = 1	n = 1	4

amitriptyline	cholinergic & serotoninergic	[65]	n = 1	n = 1	5

venlafaxine	serotoninergic & noradrenergic	[66]	n = 1	n = 1	2


In bold: discussed in the article.

As per ocular flutter, the January 2024 search retrieved three articles, with ocular flutter induced by vidarabine, toluene and cyclosporine A, the latter two being also described in toxic- or drug-induced opsoclonus.

#### Bark scorpion poison [15,16]

The scorpion responsible for this toxicity is Centruroides sculpturatus, which is light brown and commonly found in the Sonoran Desert in the southwestern United States (Arizona) and north-western Mexico. Opsoclonus is described to be prevalent after a sting, affecting both children and adults. Our literature review yielded two articles highlighting a total of n = 117 cases associated with this toxic envenomation, making it the most frequent cause of toxin-induced opsoclonus [15,16]. However, a critical evaluation is needed: from the authors’ description of the two cited papers (both articles are from the same group of authors), despite the use of the term ‘opsoclonus,’ confirmation of its high prevalence shortly after a sting remains uncertain due to the lack of consistency in the description of opsoclonus.

Potentially, the venom from C. sculpturatus leads to sodium channel inactivation and reduces the threshold for membrane depolarization, resulting in prolonged action potentials and repetitive axonal firing [15]. According to O’Connor et al. this process could increase the release of acetylcholine and norepinephrine, potentially triggering cranial nerve dysfunction, which may present as opsoclonus, tongue fasciculations, loss of pharyngeal muscle control leading to difficulty swallowing, and stridor [15,16]. The authors also suggest that these effects, combined with hypersalivation, could pose a threat to airway integrity.

O’Connor et al. [15] describe bark scorpion envenomation in 88 children, mean age 3.7 years (range 4 months-12 years) managed without antivenom therapy. 96% of them developed an opsoclonus (n = 84). Other clinical sign was motor hyperactivity (100%), tachycardia (82%), hypersalivation (81%), hypertension (49%), vomiting (38%), respiratory distress (33%), fever (28%), hypoxemia (18%), stridor (17%), and diaphoresis (11%). The mean time to symptom onset was 20 min (range 0–130 min). The mean time to health care facility was 79 min (range 10–240 min) and mean length of observation was 28.7 h (range 2–69 h). Adrenergic findings were significant, with tachycardia present in most patients, as well as hypertension and fever.

Envenomation continues to be a major public health concern in the desert southwest, especially for young children. In 2010, there were 3,498 visits to emergency departments in Arizona specifically related to scorpion stings, with the highest number of visits observed in young children [15].

In their 2018 article, O’Connor et al. report severe bark scorpion envenomation in 33 adults. Interestingly, opsoclonus remains among the most prevalent symptoms, being the second most common (n = 27), with an incidence rate of 82% [16]. Other clinical manifestations include pain/paraesthesias (n = 31), excessive motor activity (n = 25), other visual disturbance (n = 25), hypertension (n = 16), difficulty ambulating (n = 15), hypersalivation (n = 13), tachycardia (n = 12), difficulty swallowing (n = 8), vomiting (n = 5).

#### Chlordecone [17,18,19,20]

Chlordecone, also known as kepone, merex, or curlone, is an insecticide that was extensively used in the late twentieth century. However, its production and usage are now prohibited due to its numerous side effects. It is the first toxic substance mentioned in the literature to cause opsoclonus as an adverse effect. Chlordecone appears to inhibit mitochondrial Na+/K+ ATPase, which disrupts intracellular calcium transport. These alterations seem to be linked to the severity of seizures observed in cases of acute intoxication [21]. Within our literature review, four articles describe a group of workers potentially affected by this organochlorine compound [17,19,20].

The cohort of workers exposed to chlordecone comprises four articles. The first article presents a case series involving 133 workers who were professionally exposed to chlordecone in a plant manufacture between November 1973 and July 1975 [17]. The second article, published in 1978, focuses on describing the epidemiology of the exposure [18]. Subsequently, two other follow-up studies were published in 1982 and 1985 [19,20].

In their first paper, the authors reported opsoclonus in 15/133 (11%) employees. Additional signs and symptoms included tremor (n = 21), hepatosplenomegaly (n = 9), skin rash (n = 7), mental changes (n = 5) and widened gait (n = 5).

Later, Taylor et al. [19,20] report that 15 employees of the same cohort reported hereabove complained of visual disturbances, which is the same number of opsoclonus observed in reference 22; therefore, these are likely to be the same cases. Other symptoms included tremor (n = 23), chest pain (n = 18), arthralgia (n = 14), mental changes (n = 13), weight loss (n = 10), headache (n = 9), gait difficulties (n = 6) and skin rash (n = 6).

Investigators found that all employees, whether symptomatic or not, had detectable blood levels of chlordecone. Specifically, the mean level in symptomatic individuals was 2530 ng/mL, much higher than the 600 ng/mL found in asymptomatic employees. Exposure occurred through oral, respiratory, and cutaneous routes, with the authors postulating that the latter might potentially be the worst.

Taylor et al. report that “patients showed irregular burst of small-amplitude or large-amplitude saccades, usually in horizontal but also in oblique directions. These chaotic bursts of saccades were both conjugate and disconjugate and resembled opsoclonus”. Opsoclonus decreased 3 to 6 months after cessation of exposure to chlordecone but did not completely disappear (as visual disturbance was still present 4 years after) [17,19,20].

It has been observed that opsoclonus and tremulousness in this study with chronic chlordecone exposure is similar to rapid eye movements and limb tremors observed in dogs infused with the drug tremorine, which is a cholinergic drug not commonly used as therapeutics but primarily used in research, whose site of action is the mesencephalic reticular formation [22]. Thus and by analogy the site of action for chlordecone might be similar [17].

In the 1982 follow-up [19], 23 patients (out of the 133 examined in 1978) still presented active symptoms at the time of the evaluation. The cardinal symptom of chlordecone intoxication, 4 years after the cessation of exposition, was tremor (n = 23) following by chest pain (n = 18), visual disturbance (n = 15), mental changes (n = 13), weight loss (n = 10), headache (n = 9), gait difficulty (n = 8), rash (n = 6), and arthralgia (n = 4). Examination of clinical signs revealed tremor (n = 21), ocular flutter/opsoclonus (n = 15), hepato/splenomegaly (n = 9), rash (n = 7), mental changes (n = 5), and widened gait (n = 5).

Subjects noted “brief periods of visual blurring, particularly upon looking quickly to one side or the other” and observations confirmed that opsoclonus was still present in all 15 cases four years after cessation of exposition [19].

In terms of pathophysiology the authors postulate that chlordecone-induced ocular motility disturbance does not interfere with the normal function of the fronto-pontine saccadic pathway for conjugate saccades [17]. They assume that chlordecone might cause opsoclonus by suppressing “pause cells” (probably referring to the omnipause neurons, a/n) by increasing post-inhibitory rebound excitation [19].

#### Diphenhydramine [23,24,25]

Diphenhydramine is an anticholinergic and antihistaminic drug indicated in allergies, insomnia and common cold. Three articles of our literature review mention diphenhydramine causing an opsoclonus as a complication of overdose [23,24,25].

Those three case reports mention three subjects (20- and 36-year-old women and 22-year-old man) having ingested diphenhydramine in a suicidal attempt. The drug ingestion quantity was 5g [25], 3.3g [23] and 2g [24]. Opsoclonus onset occurred between 2 to 10 hours post ingestion [25] to several days [23,24]. Serum level of diphenhydramine was only tested in one case being 2.61 μg/mL [23]. Opsoclonus resolved by 8 hours after arrival at the health care facilities for the 20yo woman, after she underwent endotracheal intubation for airways protection, and was treated with sodium bicarbonate [25]. Opsoclonus disappeared on the second hospital day for the 22yo man as he received IV fluids and 800mg valproic acid daily [23]. Both opsoclonus and ataxia improved in the 36yo patient after introducing clonazepam (3mg/day) [24]. Associated symptoms for the three cases consisted in: generalized seizures and sinus tachycardia [25]; coma, seizure, autonomic disturbance, rhabdomyolysis and mental status changes [23] and agitation, confusion with uncoordinated limb movement [24].

#### Organophosphates (OPPs) [26,27,28]

Organophosphate is a group of approximately 40 pesticides that kill insects and other animals by impacting the function of central and peripheral nervous system by inhibiting the activity of acetylcholinesterase (AChE) [29].

Our literature review emphasizes that OPPs might cause opsoclonus. We identified three articles that mention three different cases (n = 3) of opsoclonus caused by organophosphate ingestion, accidental or during a suicide attempt [26,27,28]. The different OPPs described are: chlorpyrifos [26], malathion [27] and an insecticide containing parathion and methyl-parathion [28].

In these three case reports are described two 29-year-old men, one with chronic paranoid schizophrenia (treated daily with flupentixol 20mg, procyclidine, thioridazine and flurazepam) and one with no particular comorbidity, who ingested an unmentioned amount of OPP in a suicide attempt [27,28]. The third case describes a 12-year-old girl who accidentally ingested OPP [26].

All three cases required urgent intervention. Main manifestations on admission were cholinergic crisis symptoms like vomiting, hypersalivation, associated with lingual myoclonus, ptosis, somnolence, areflexia, subfebrile state and tachypnoea, even leading to respiratory arrest for the 12-year-old girl [26]. The two 29-year-old men had a slightly less severe presentation with drowsiness, unresponsiveness to painful stimuli, cyanosis, with typical cholinergic symptoms including profuse fasciculations and muscle cramps [27,28].

The opsoclonus occurred after 2 or 3 days of hospitalization in all three patients and improved after about 2 weeks [26,27,28]. Management was in all three cases consisted of atropine and oximes (common treatments of organophosphate intoxications [30]), and additionally with antibiotics [31], intubation and ventilation [27], gastric lavage and oral mannitol [28]. The hypothesized pathophysiology [27,28] was that inhibition of AChE caused by OPPs, induced a high level of synaptic cleft acetylcholine in the tectal/pretectal areas and in the midbrain tegmentum, thus causing opsoclonus [27].

#### Antiepileptics [32,33,34]

Antiepileptic drugs are a diverse group of pharmacological agents used in the treatment of epilepsy. They are also being increasingly used in the treatment of various neuro-psychiatric and neurologic conditions, like bipolar disorder, borderline personality disorder, acting as mood stabilizers, and for the treatment of painful neuropathies. Common antiepileptic drugs block voltage-gated Na-channels or enhance GABA function, although several antiepileptic drugs have other or multiple or even uncertain mechanisms of action.

We describe three different cases (n = 3) of opsoclonus reported as an undesirable effect of an antiepileptic drug, probably phenytoin in 2 cases and possibly lamotrigine in the remaining case [32,33,34]. Due to the uncertain mechanism of action and specific role of each antiepileptic drug, these cases are going to be featured separately.

Thome-Souza et al. [32] describe a 6-year-old girl with a 42-month history of partial symptomatic epilepsy, characterized by partial motor seizures, which had become refractory to treatment. The patient was brought under control with a combination of lamotrigine (200 mg/day), valproate (90 mg/day), and nitrazepam (19 mg/day) when she was 4 years old. Nine months later she presented episodes of vomiting, approximately once every 15 days. They excluded infection or metabolic imbalance. EEG was unaltered. She presented with tremor and dizziness, and one month later she was clinically evaluated with an opsoclonus characterized by “irregular, nonrhythmic, agitated oscillations of the eyeballs in both the horizontal and vertical planes”, together with ataxia, vomiting, headache, and vertigo. They decreased by 50 mg daily the dose of lamotrigine (bringing it to 150 mg/day) for 2 weeks, keeping valproate at the same dosage, which led to the remission of these disturbances without worsening the seizures. Therefore, lamotrigine might have caused the opsoclonus. Thome-Souza and al. [32] stated that “the mechanisms of movement disorders are highly hypothetical, and antiepileptic drugs have been postulated to affect Na+ channels, acting on dopaminergic metabolism through an inhibition of glutamine. Abnormal eye movements were also hypothesized to be caused by increased epileptiform discharges”.

The second case is featured by Dehaene and al. [33] about a 44-year-old woman presenting a tonic-clonic seizure 24 hours after hysterectomy, followed by a second seizure 12 hours later. The seizures might have been precipitated by the abrupt withdrawal of long-term analgesics containing barbiturates. Phenytoin and diazepam were administrated intravenously, respectively 750 mg (50 mg/min) and 10 mg, while neurological findings were normal. One hour later she developed slurred speech, marked ataxia, and noted oscillopsia shortly after phenytoin infusion. “The eyes made rapid conjugate involuntary movements in all directions. Electro-oculography was performed, revealing double saccadic pulses and bursts of back-to-back saccades without saccadic interval, both in the horizontal and vertical planes” [33], corresponding to the definition of opsoclonus. Phenytoin serum level was 19.3 μg/ml one hour after the start of the phenytoin infusion, and cerebrospinal fluid analysis came normal. “Opsoclonus, oscillopsia, dysarthria, and ataxia disappeared spontaneously after approximately two hours”. They performed an EEG, and a brain CT on the next day and both revealed normal findings. The authors state that barbiturate withdrawal may have played a role, as well as diazepam infusion, although the latter is less probable, as some benzodiazepines can diminish opsoclonus [35]. They point the omnipause cells of the pontine paramedian reticular formation as a key structure for the development of opsoclonus [33].

Verma et al. [34] reported a 19-year-old male presenting a 12-day history of abnormal, episodic intermittent involuntary jerky movements of all four limbs, trunk, and eyelids without loss of consciousness, and gait difficulties with swaying. Clinical findings revealed “abnormal chaotic multidirectional movement of eyes suggestive of opsoclonus with evidence of myoclonic jerks involving all four limbs, face, and eyelids (…) as well as gait ataxia, incoordination and dysmetria” [34] evoking an OMAS. He recently had an increase in antiepileptic drug regimen (phenytoin was increased from 300 to 500 mg/d and levetiracetam 500 mg BD was added) [34]. As a corelate, serum phenytoin was markedly high (>40 μg/ml). Verma and al. [34] assumed that it was provoking the opsoclonus because as phenytoin was stopped, levetiracetam 500 mg bd maintained and clonazepam 0.5 mg tds was started, all his symptoms including OMAS gradually disappeared over next 15 days [34]. Moreover, OMS had appeared after recent increment of dose of phenytoin, a drug known for its narrow therapeutic window [34].

#### Cocaine [36,37]

Cocaine is a tropane alkaloid and central nervous system (CNS) stimulant drug consumed for its euphoric and rewarding effects. It has several effects principally blocking transporters that reuptake neurotransmitters: it blocks dopamine, serotonin, and norepinephrine transporters, thus leaving more of these compounds in the synaptic cleft and increasing activation of their receptors in the post-synaptic neurons.

We identified two different cases from two different articles of cocaine-induced opsoclonus [36,37], illustrating acute cocaine intoxication of a woman and a man in their 30s. Both cases report severe and uncontrollable shaking of the entire body, causing difficulty in standing, nausea and vomiting. The case reported on admission by Elkardoudi-Pijnenburg et al. [36] was tachycardic, normopneic near the upper limit of the norm (20/min), normotensive and afebrile. On exam the eye movements were described as crescentiform with coarse eye movements in all directions with a frequency of 8 Hz. As for Scharf et al. [37], the case was reported as having marked continuous opsoclonus consisting in spontaneous conjugate jerking observed in all directions, as well as in a clockwise rotatory direction.

In both cases the eye movement disorder improved gradually, Elkardoudi-Pijnenburg and al. [36] describing an evolution from opsoclonus to flutter-like oscillations, and to sporadic horizontal ocular myoclonus in vertical movements. The four months follow up revealed no more oculomotor abnormalities nor ataxia.

#### Cyclosporine A (CsA) [7,38]

Cyclosporine is a calcineurin inhibitor, used as an immunosuppressant medication. Two articles describe an opsoclonus induced by this drug [7,38]. It has a well-known neurotoxicity probably due to its highly lipophilic nature allowing it to cross blood-brain barrier and provoking change to neurotransmission through altered dopamine receptor function [7,38,39].

Eurípedes-Marchiori et al. [7] describe a 17-year-old female who underwent a liver transplantation for an autoimmune cirrhosis. The immunosuppression was CsA (10 mg/kg/d), prednisone 20 (mg/d) and azathioprine (75 mg/d). Eight days after the transplant she developed an opsoclonus and generalized seizures, in the setting of a reversible posterior leukoencephalopathy. Analysis of the CSF was normal, and MRI revealed bilateral hyperintensities in occipital lobes on T2 weighted sequences. The ocular symptoms improved 21 days after the reduction of the CsA level, while keeping the other immunosuppressors at the same dosage.

Kang et al. [38] describe an opsoclonus induced by cyclosporine as a long-term use side-effect . A 37-year-old woman had been taking CsA (200 mg/day) and prednisolone (5 mg/day) for 17 years following a kidney transplant, when she presented a 2-weeks history of dizziness, opsoclonus, sometimes only ocular flutter and mild myoclonic movements in her upper extremities. The patient reported a flu-like episode three weeks prior to the onset of OMS and had slept very little for three days. Brain lesions were excluded based on MRI and angiography. CSF, blood test and CT scans (chest, abdomen, and pelvis) were all normal. Tumor, autoimmune and paraneoplastic markers were negative. CsA was replaced by tacrolimus two weeks after the onset of symptoms, which was followed by gradual improvement of opsoclonus one week later.

Eurípedes-Marchiori and al. [7] argue that the opsoclonus of the liver transplanted patient occurred because of neurotoxicity of CsA and probably because of metabolic or neurotransmitter abnormalities of omnipause cells.

In a 17-year-old female diagnosed with acute myeloid leukaemia, Cyclosporin A was found to induce ocular flutter, as reported by Apsner et al. [40]. The drug was administered as a prophylaxis against graft-versus-host disease, and clinical symptoms resolved within three weeks after discontinuation of CsA.

#### Toluene (Methylbenzene) [41,42]

Toluene, also known as toluol, is a substituted, colourless, water-insoluble liquid aromatic hydrocarbon associated with paint thinners. This solvent is sometimes used as a recreational inhalant for its drunken-type actions. It has been banned from many industries and its production is regulated for its potential of causing severe neurological harm, such as opsoclonus as we are going to emphasize through two cases reports [43]. Toluene exerts its effects by inhibiting excitatory ion channels like the NMDA and nicotinic receptors, while enhancing the function of inhibitory ion channels such as the GABA-receptor type A, glycine, and serotonin receptors. Additionally, toluene disrupts voltage-gated calcium channels and ATP-gated ion channels [44].

Lazar et al. [41] report a case of a 25-year-old man who has a 5-year history of massive and day-long toluene abuse. “He awoke in the morning and sniffed a rag soaked with toluene from morning until night, removing it only to void or eat” [41]. He noted for 4 years progressive tremor of the limbs, trunk, and head; difficulty maintaining balance; slurred speech; bilateral hearing loss; and continuous jerking movements of the eyes. Clinical findings included a full cerebellar syndrome with ataxic gait, four-limb ataxia, scanning speech and head and truncal titubation. Rapid, involuntary, repetitive, chaotic, conjugate, saccadic eye movements were noted in all directions, typical of opsoclonus, as per the authors [41]. CT revealed a moderate atrophy of cerebellar cortex, cerebellum, and brainstem. In this study from Lazar et al. [41], opsoclonus was present in one patient and ocular flutter in two out of the four subjects.

Brainstem auditory evoked potential testing revealed normal latency for wave I, but absolute latencies for waves II,III, IV, and V were three standard deviations above the mean for normal age-matched subjects [41].

Papageoriou et al. [42] present a 22-year-old man who had been sniffing toluene for the past 10 years on a daily basis. Initial clinical symptoms began 2 years before the consultation, with bilateral tremor. Several other manifestations then developed such as ataxic gait and speech deterioration. Clinical examination revealed confusion, drowsiness, opsoclonus, dysarthria and tetraparesis, and MMSE score was 6/30. Brain MRI, T2-weighted images (1.5 T) showed mild cerebral and cerebellar atrophy, slightly increased signal in white matter and cerebellum and low signal bilaterally in many brainstem, cerebellar and basal ganglia nuclei on both sides (dentate, red, substantia nigra, thalamus, hypothalamus, caudate, globus pallidus and putamen). Single photon emission computed tomography (SPECT) with 123I Ioflupane (123I-FP-CIT or DaT-scan) showed a bilateral decrease in presynaptic dopamine reuptake [42], confirming nigro-striatal degeneration.

#### Lithium associated with haloperidol [45]

Lithium salts are used as a psychiatric medication, classified as mood stabilizers, primarily for bipolar and for major depressive disorders. It is believed that it interacts with number of neurotransmitters and receptors, decreasing norepinephrine release and increasing serotonin synthesis. Haloperidol is a typical antipsychotic medication used in many disorders such as schizophrenia, mania in bipolar disorder, delirium, agitation, acute psychosis, and hallucinations. It has a high-affinity for D2-receptor antagonism [46]. Even though these drugs have many known side effects, opsoclonus is commonly not listed in. Two cases of opsoclonus potentially induced by the combination of these medications have been reported.

Cohen et al. [45] describe two patients with diagnoses of mania treated with a combined regimen of lithium and high dose of haloperidol. In the first case haloperidol then lithium was introduced, but then patient became stuporous. Haloperidol and later lithium were both discontinued and there was mention of intermittent opsoclonus in the ten-month follow-up. In the second case, lithium and haloperidol were introduced together for a manic episode, which led to confusion and lethargy. It seems that the opsoclonus appeared around a week after drug discontinuation, persisting up to ten months after admission. Detailed chronology is missing and contribution of either of these two drugs to the development of opsoclonus cannot be ascertained, hence a low Naranjo score of four.

#### Phencyclidine (PCP) [9]

Also called phenylcyclohexyl piperidine, or know as angel dust, it is a drug initially produced as an anaesthetic, but nowadays mainly used recreationally for its significant mind-altering effects. PCP has many actions on the CNS. It is a non-competitive NMDA receptor antagonist that can increase non-NMDA glutamatergic transmission. It inhibits GABAergic output. It decreases dopamine and norepinephrine uptake, and increases the dopamine and norepinephrine levels by stimulating the enzyme tyrosine hydroxylase [9,47,48].

Shameer Nijam and al. [9] describe a 21-year-old male with a 3-day history of headache, decline in overall well-being, aggressiveness and agitation who developed generalized seizures before admission. The patient was not on prior medication and presented diaphoresis, hypersalivation, and at times ocular flutter and opsoclonus. He was tachycardic (140–160 beats/min), hypertense (170/100 mmHg), and pyretic (40°C), His liver enzymes were disturbed (AST 350 IU/L; ALT 153 IU/L) and CK was high suggesting rhabdomyolysis (40,000 μg/l). Toxicological urine screening was positive for PCP. Despite the treatment he rapidly deteriorated and passed away due to an acute renal failure secondary to rhabdomyolysis.

The authors propose that the link between PCP and opsoclonus is due to an imbalance between GABA and glutamate, leading to the disinhibition of either omnipause neurons in the pontine nucleus raphe interpositus or neurons in the FN. Both the pontine nucleus raphe interpositus and fastigial nucleus are primarily regulated by glutamate and GABA, and the high doses of PCP may lead to their disinhibition. This could give rise to ocular flutter or opsoclonus [5,49].

#### Solanum torvum berries [49]

Also known as turkey berries, susumber, pea eggplant or turkey berry, this plant grows essentially under the tropics and its berries are traditionally cooked in various cultures [49]. It contains various glycoalkaloids that inhibit acetylcholinesterase and indirectly activate cholinergic receptors with varying degrees of potency. Clinical symptoms are therefore likely the result of concomitant muscarinic and nicotinic stimulation [49,50,51].

Glover et al. [49] describe a 54-year-old woman presenting at the Emergency Department with complaints of gait, vision and speech changes, vomiting and diffuse myalgias since the same morning, suggestive of cholinergic crisis. Physical examination revealed: intact, lucid mental status, miosis, opsoclonus, severe dysarthria, dysmetria, mild extremity tenderness and weakness, and inability to ambulate. Her vital signs were: 168/88 mm/Hg; 83 beats/min; 14 resp/min; 36.8°C, 100% oxygen saturation. Serum creatinine phosphokinase was measured twice: 350 IU/L; 12 hours later: 1844 IU/L. She reported eating a meal including many boiled susumber berries, on her own. Supportive care was administered, and neurological deficits resolved gradually as she was discharged on day three. She reported no recurrence of the symptoms one month later.

#### Thallium [52]

One single conference abstract mentions a case of thallium poisoning in a teenager attempting to suicide, who among other symptoms developed opsoclonus [53]. Thallium was notably used as a pesticide in the 20’s. Its toxicology relates to interference with the vital potassium-dependent processes, for instance substituting itself to potassium in the (Na+/K+)-ATPase [52].

#### Cefepime [54]

Cefepime is a fourth-generation cephalosporin antibiotic and has an extended spectrum of activity against Gram-positive and Gram-negative bacteria. Cephalosporins might produce reversible neurotoxicity due to GABA receptor antagonism, particularly when administered during renal dysfunction [54].

Lizarraga et al. [54] report a 70-year-old woman without any significant medical history presenting with severe headache. Neurological examination was normal, but investigations revealed a subarachnoid haemorrhage due to a leaky aneurysm that was immediately coiled. Four weeks later she presented persistent and unexplained delirium. Meningitis was suspected as CSF analysis revealed elevated leucocytes with 95% lymphocytes. Vancomycin and cefepime (2g/8h) were administrated intravenously. Three days after antibiotic initiation her mental status deteriorated to coma, and she developed opsoclonus with multifocal stimulus-sensitive myoclonus. Continuous video-EEG monitoring demonstrated a generalized, 2–3 Hz triphasic wave pattern, without clear electrographic seizures. Lorazepam and levetiracetam resolved the triphasic pattern. As CSF culture were reported negative, antibiotics were discontinued, and her mental status improved. Levetiracetam was also discontinued and opsoclonus resolved.

Cefepime commonly presents with encephalopathy and myoclonus [55,56]. It is probable that the high dose of this cephalosporin administrated after a recent brain injury had predisposed this patient to neurotoxicity despite a normal renal clearance [54].

#### Vidarabine [57]

A 29-year-old woman with acquired immunodeficiency syndrome, exhibited ocular flutter, myoclonus, ataxia, and mild confusion six days after intravenous vidarabine treatment for severe genital herpes simplex infection. Electro-oculography revealed horizontal saccadic oscillations, diminishing two days post-therapy cessation. The patient’s neurological examination normalized the following day, emphasizing the association between vidarabine and the neurological syndrome, including ocular flutter. CSF examination excluded CNS infection. The authors mention an autopsy study in vidarabine toxicity, which revealed neuronal chromatolysis, suggesting metabolic injury, in various brain regions such as cerebral cortex, the basal ganglia, the thalamus, the brainstem, and the dentate nucleus of the cerebellum.

## Discussion

Opsoclonus is a disorder of the saccadic system that can result from several different etiologies. Here, we present the case of a patient with multiple system atrophy (MSA) in whom opsoclonus was most likely induced by amantadine, serving as the impetus for conducting a comprehensive review of toxic/drug-induced causes of opsoclonus.

As amantadine (and phencyclidine) acts as an NMDA-R antagonist, we searched and found articles reporting opsoclonus in patients with anti-NMDA-R encephalitis [58,59], further supporting the possibility that amantadine, in our case, may have indeed caused the opsoclonus. However, it is important to note that a substantial series of 249 cases with ovarian teratoma, which predominantly involved antibody-positive NMDA-R encephalitis (n = 211), revealed opsoclonus in 10 out of the 38 NMDA-R seronegative cases (27%), whereas none of the 211 antibody-positive cases exhibited opsoclonus [60]. This indicates that the relationship between NMDA-receptor dysfunction and opsoclonus cannot be firmly established.

Regarding any potential link between MSA and opsoclonus, we found only two articles [61,62] in the literature reporting opsoclonus in MSA patients, but no specific etiology was identified despite thorough investigations. Thus, it is unlikely that the opsoclonus in our case is related to this neurodegenerative condition. Additionally, the absence of opsoclonus recurrence during the three-year follow-up further diminishes the likelihood of a direct association between MSA and opsoclonus. Moreover, the administration of amantadine on two distinct occasions, several months apart, each resulting in the development of opsoclonus, strongly suggests that amantadine is the causative agent. Consequently, amantadine can be included to the list of drugs and toxins known to induce opsoclonus, although extremely rarely.

Speed of onset considerably varied between the different agents, with opsoclonus developing sometimes within minutes or hours (bark scorpion poisoning, diphenhydramine, phenytoin-intoxication/versus-barbiturate withdrawal), days (amantadine, organophosphates, diphenhydramine, cyclosporin A, phenytoin, cefepime) or months/years (cyclosporin A, lamotrigine, chlordecone). Similarly, rate of improvement considerably varied with opsoclonus resolution within days (diphenhydramine, phenytoin-intoxication/versus-barbiturate withdrawal, amantadine), weeks (organophosphates, cyclosporin A, phenytoin) or persisting for months/years (chlordecone, combination of lithium and haloperidol). For the latter however, with persistent opsoclonus 10 months after lithium and haloperidol discontinuation, the low imputability of these drugs (Naranjo score of four) to opsoclonus must be taken into consideration before drawing conclusions.

No common mechanism of action was identified among the various drugs listed in Table 1 that trigger opsoclonus. It appears that opsoclonus can be initiated by a variety of imbalances or dysfunctions affecting different neurotransmitter systems, ionic channels, or ionic pumps, particularly at the various synapses illustrated in Figure 1, adding fuel to the mill of the “pontine/brainstem theories”. Association of opsoclonus with cerebellar dysfunction, notably in the setting of OMAS, or in some of the cases reported (e.g. toluene, phenytoin, diphenhydramine intoxications), points to the “cerebellar theory” described above, with dysfunction of Purkinje cells or Fastigial nucleus.

The most commonly described agent, accounting for three-quarters of reported cases, is the bark scorpion poison, which inactivates voltage-gated sodium channels and possibly increases the release of acetylcholine. This neurotransmitter is also implicated in other drugs causing opsoclonus, like organophosphates, diphenhydramine (an anticholinergic), and solanum torvum berries; cholinergic crisis however only infrequently causes opsoclonus. Interestingly there was an extremely high proportion of patients suffering from bark scorpion stinging who developed opsoclonus, respectively 82% and 96% of the adults/children reported. The second most common agent triggering opsoclonus is chlordecone, an insecticide that inhibits the mitochondrial Na+/K+ ATPase, with 15 cases reported.

It is worth noting that in articles discussing these two toxins, there are inconsistencies in defining opsoclonus. O’Connor et al. [15,16] describe opsoclonus resulting from bark scorpion venom poisoning as “rapid, irregular, multidirectional, and dysconjugate eye movements,” while Martinez et al. [17] characterize the chlordecone-induced opsoclonus as “irregular bursts of small-amplitude or large-amplitude saccades, usually in horizontal but also in oblique directions (…) both conjugate and dysconjugate and resembled opsoclonus.” It’s important to note that these descriptions don’t precisely match the established definition of opsoclonus, which specifically involves conjugate eye movements.

Additionally, the absence of mention of bark scorpion in Zee and Leigh’s textbook [1] (table 11-8) raises doubts about whether it truly represents cases of opsoclonus. Therefore, addressing this gap in the literature is crucial, prompting a further exploration of the relevance and validity of the association between opsoclonus and bark scorpion venom poisoning.

The remaining cases involve various neurotransmitters, and it appears that perturbations in most neurotransmitter systems have the potential to trigger opsoclonus. However, the extreme rarity of drug-induced opsoclonus must be considered alongside the high number of putative mechanisms and the frequent use of some of the listed drugs (e.g., diphenhydramine). This complexity prevents us from drawing precise conclusions regarding the pathophysiology of opsoclonus. Additionally, the fact that opsoclonus can persist for years after the cessation of the offending agent (e.g., chlordecone) raises further questions about its underlying mechanisms.

### Treatment of opsoclonus

Etiologic treatment is preferred, when available, e.g. in the case of opsoclonus triggered by brainstem encephalitis or infectious agents [2]. As per symptomatic treatments, there is no evidence-based medicine, but a few reports, with rather modest benefits, stating the use of clonazepam, propranolol, gabapentin, or topiramate, with the objective to enhance GABAergic transmission of Purkinje cells [2]. In the latest 2015 version of Zee & Leigh “neurology of eye movement”, several putative treatment options are mentioned, based on the different ionic channels at the surface of saccadic burst neurons and drugs acting on their binding sites (figure 11-24-C). [1].

### Limitations

As often observed in medical literature, many articles refer to other articles, creating a chain of citations. However, digging deeply to find the original source can sometimes be disappointing. A notable example is the case of strychnine poisoning, where the referenced article is cited in various publications, including the beautiful textbook by Leigh and Zee [1]. However, the original article is merely a brief abstract that does not mention the term “opsoclonus” and describes a “horizontal pendular nystagmus,” which is inconsistent with the definition of opsoclonus. Therefore, including strychnine in the list of agents triggering opsoclonus may not be justified.

Regarding thallium, it is essential to note that the only mention of it causing opsoclonus comes from a brief conference proceeding. Nevertheless, the description of “prominent chaotic eye movements” and the use of the term “opsoclonus” in this report make the association somewhat convincing. However, we should consider the fact that thallium was commonly used as a pesticide for more than 40 years in the 20th century, and there are hundreds of articles about thallium poisoning in PubMed. This extensive literature on thallium poisoning should be carefully considered in light of this single short report of thallium-induced opsoclonus.

It is also important to emphasize that, during our literature review, we observed that certain articles mentioned opsoclonus without adhering strictly to its definition, as discussed hereabove. This variability in the application of the term necessitates caution in interpreting and generalizing findings related to opsoclonus in the context of the discussed toxins.

Additionally, in some articles of our literature review [49,50,52,63,64], only the term opsoclonus or OMS is mentioned without providing a detailed description of the ocular disturbance or a clear temporality [45], or present a video of eye movement not compatible with opsoclonus [64]. This lack of specific details makes it challenging to ascertain whether the reported condition truly corresponds to an opsoclonus.

Finally, multiple drugs being prescribed concomitantly (e.g.: in the case of the antiepileptics described hereabove), it is not always possible to draw precise conclusions as per which drug caused the opsoclonus.

## Conclusion

In a nutshell, opsoclonus is a multifaceted disorder involving multiple systems and neurotransmitters responsible for eye movement stabilization and coordination. Dysregulation of neurotransmitters like acetylcholine, glutamate, GABA, dopamine, and glycine, along with drugs affecting sodium channels, can disrupt the precise neural activity timing, leading to the irregular and uncontrollable saccadic eye movements observed in opsoclonus.

Considering the various drugs and toxins identified in this review, it is conceivable that polymedication may also play a significant role in causing or exacerbating opsoclonus in cases with more typical etiologies. Interactions between different medications, each affecting neurotransmitter systems or ionic channels, can lead to imbalances and dysregulation, contributing to opsoclonus development.

While autoimmune and paraneoplastic encephalitis, as well as other central nervous system pathologies and infections, remain the most common causes of opsoclonus, it is important to acknowledge that toxins and drugs, although extremely rare culprits, also have the potential to induce opsoclonus, complicating our understanding of the complex pathophysiology of this condition.

Based on the findings of this study, we propose that amantadine should be added to the list of medications potentially causing opsoclonus.

Healthcare professionals must remain vigilant in monitoring the effects of medications, especially when multiple drugs are used concurrently. This cautious approach is crucial for identifying any adverse interactions that might contribute to the onset or exacerbation of opsoclonus. By better understanding the underlying mechanisms and risk factors, we can improve patient care and enhance the management of this rare but intriguing disorder.
